# Supplementary material for: Decline of Lung Function in Knee and Spine Osteoarthritis in the Korean Population: Cross-Sectional Analysis of Data from the Korea National Health and Nutrition Examination Survey
Source: Healthcare (Basel). 2022 Apr 15;10(4):736. doi: 10.3390/healthcare10040736 (PMC9027391; doi:10.3390/healthcare10040736)
Supplement: Supplementary file 1 [file healthcare-10-00736-s001.zip › healthcare-1655875-supplementary.pdf]

Supplementary

Table S1. Multivariate-adjusted ORs (95% CIs) for variables related with the presence of OA

Parameters	Knee OA			Spine OA		
	OR	95% CI	p value*	OR	95% CI	p value*
Age	1.111	1.091 – 1.132	<0.001	1.091	1.068 – 1.115	<0.001
Sex (ref: female)	0.483	0.329 – 0.709	<0.001			
Body mass index	1.193	1.145 – 1.242	<0.001			
Smoking (ref: non-smoker) †						
Ex-smoker	0.660	0.435 – 1.001	0.849			
Current smoker	0.950	0.562 – 1.607	0.153			
Alcohol consumption (ref: non-alcoholic) †						
Alcoholic	0.982	0.703 – 1.373	0.916	1.002	0.700 – 1.434	0.990
Hypertension (ref: none) †	1.315	1.028 – 1.683	0.029	1.255	0.923 – 1.707	0.146
Bronchial asthma (ref: none) †	1.953	1.102 – 3.460	0.022			
Myocardial infarction/angina (ref: none) †				1.548	0.783 – 3.061	0.207
Diabetes mellitus (ref: none) †				0.365	0.907 – 2.055	0.135
COPD (ref: none)				0.995	0.672 – 1.474	0.979

Abbreviation: OR, odds ratio; CI, confidence interval; OA, osteoarthritis; COPD, chronic obstructive pulmonary disease

*p values were obtained by the composite sample multivariate logistic regression analysis.

†Missing data were excluded from the analyses: for smoking, n = 19; for alcohol consumption, n = 18; comorbidities (hypertension, myocardial infarction/angina, bronchial asthma, and diabetes mellitus), n = 16.

Table S2. Comparison of lung functions between OA with both knee and spine and controls

	Controls (n = 1198)	Knee OA (n = 414)	Spine OA (n = 394)	OA with both knee and spine (n = 323)	*p* value (Control vs. both knee and spine OA)	*p* value (Knee OA vs. both knee and spine OA)	*p* value (Spine OA vs. both knee and spine OA)
Spirometry							
FVC (L)	3.56 (0.03)	3.18 (0.05)	3.41 (0.06)	2.93 (0.05)	<0.001	<0.001	<0.001
FVC (%)	92.2 (0.48)	91.1 (0.6)	92.3 (0.7)	91.62 (0.77)	0.824	0.174	0.902
FEV_1 (L)	2.71 (0.02)	2.42 (0.04)	2.52 (0.05)	2.19 (0.04)	<0.001	<0.001	<0.001
FEV_1 (%)	90.3 (0.5)	91.9 (0.8)	91.0 (1.0)	94.13 (0.97)	<0.001	0.025	0.002
FEV_1/FVC (%)	0.76 (0.00)	0.76 (0.00)	0.74 (0.01)	0.75 (0.01)	0.001	0.022	0.391

Abbreviation: FVC, forced vital capac1ity; FEV_1, forced expiratory volume in one second; COPD, chronic obstructive pulmonary disease; OA, osteoarthritis

p values were compared to controls and obtained by two sample t-test or chi-square test.
